# Effect of L-Carnitine on Muscle Quality and Antioxidant Capacity of Hybrid Sheep at an Early Stage

**DOI:** 10.3390/ani15172564

**Published:** 2025-08-31

**Authors:** Xia Qin, Wenjie Liu, Jiaqi Xin, Yidan Zhang, Mingxi Zhang, Weiwei Liang, Jiantao Li, Jianmin Hu

**Affiliations:** College of Animal Science and Veterinary Medicine, Shenyang Agricultural University, Shenyang 110866, China; qinxia@syau.edu.cn (X.Q.); 15326105139@163.com (W.L.); 18742129227@163.com (J.X.); 17624005326@163.com (Y.Z.); 2018500063@syau.edu.cn (M.Z.); liangweiwei@syau.edu.cn (W.L.)

**Keywords:** L-carnitine, meat color, sheep, ferric myoglobin reductase, antioxidant enzymes

## Abstract

L-carnitine supplementation enhances fatty acid oxidation and antioxidant defense mechanisms in livestock. Twenty-four wether lambs were randomly assigned to one of three dietary treatments: a control (basal diet), a low-L-carnitine diet (0.01%), or a high-L-carnitine diet (0.05%). After a 15-day adaptation period, all lambs underwent a 45-day experimental phase to assess the impact of L-carnitine supplementation on performance, carcass, muscle, and antioxidant capacity. Although growth performance did not show significant differences (*p >* 0.05), muscle pH increased in the L-carnitine group (*p <* 0.05), while drip loss decreased in both the low- and high-dose groups (*p <* 0.05). The a* value of the Longissimus dorsi muscle significantly increased (*p <* 0.01), enhancing meat color saturation (*p <* 0.05), with a reduction in the hue angle observed in the high-dose group (*p <* 0.01). L-carnitine supplementation led to an elevated myoglobin content and a higher proportion of oxymyoglobin, driven by a significant increase in MRA activity (*p <* 0.01). The concentration of L-carnitine was positively correlated with the a* value, which in turn was associated with higher total myoglobin content and a greater proportion of oxymyoglobin. In terms of antioxidant capacity, the L-carnitine group exhibited a significant increase in superoxide dismutase content. Moreover, there was upregulation of *TFR1* and *CAT* expression at the mRNA level, while *HEPH* showed downregulation (*p <* 0.01). Significant increases were observed in both content and mRNA expression of *LDHB* and *NADHBR5* (*p <* 0.01). The addition of 0.05% L-carnitine to the diet significantly enhanced muscle color stability in hybrid sheep. This improvement was primarily driven by a significant increase in MRA activity in the high-dose group, which facilitated the conversion of metmyoglobin to oxymyoglobin, thereby significantly boosting meat quality prospects for the sheep industry.

## Simple Summary

L-carnitine, a green feed additive, plays an essential role in the processes of muscle oxidation and reduction. However, its mechanism of influencing the color formation of lamb meat has not been fully elucidated. This research involved systematic feeding and slaughter experiments aimed at assessing the impact of dietary L-carnitine supplementation on early growth performance, slaughter outcomes, meat quality, muscle color stability, and associated gene expression in lambs. The results demonstrated that adding L-carnitine to the diet improved the redox stability of muscle tissue in early-growing lambs, enhanced the activity of the lactate dehydrogenase redox system, promoted the activity of metmyoglobin reductase (MRA), and effectively enhanced the stability and redness (a* value) of lamb meat. This study provides important insights into the use of L-carnitine as a nutritional additive in lamb diets and establishes a crucial foundation for future research on the mechanism of lamb muscle color regulation.

## 1. Introduction

L-carnitine, an essential physiological substance, plays a crucial role in regulating animal energy metabolism [1]. It facilitates the efficient transport of long-chain fatty acids to the mitochondria, thereby promoting beta-oxidation and fulfilling critical functions in maintaining energy balance and antioxidant defense [2,3,4]. With the livestock industry expanding and the demand for high-quality meat increasing, academic research has been conducted on the effectiveness of L-carnitine in improving meat quality in animals.

L-carnitine has been shown to have significant physiological regulatory effects in various animal species [5]. Studies on tilapia have confirmed that L-carnitine supplementation can enhance growth rate [6], improve biological characteristics, optimize meat moisture retention and color [7]. Research on *Trachinotus ovatus* has indicated that L-carnitine supplementation could enhance growth performance, promote lipid metabolism, and increase antioxidant capacity [8]. Experiments on largemouth black bass juveniles suggest that the combined addition of L-carnitine and nucleotides can synergistically improve growth performance and feed efficiency [9]. Similarly, research on yellowtail has confirmed the positive effects of L-carnitine on growth, body composition, and antioxidant indicators [10]. Studies in poultry farming have shown that supplementation with L-carnitine can reduce fat deposition, increase protein content, and enhance meat quality [11,12]. Research on pork quality has indicated that L-carnitine has the potential to improve muscle fatty acid composition and sensory characteristics [13,14]. However, a study on low-birth-weight -pigs found that adding carnitine and arginine to milk replacer did not significantly affect pig growth, carcass characteristics, meat quality, or muscle morphology in the long term [15]. The enzyme carnitine palmitoyltransferase (CPT) is crucial for energy regulation and lipid metabolism, with S-1-propenylcysteine being identified as a compound that can boost fatty acid metabolism by activating muscle-specific CPT [16]. Investigations on dairy cows have revealed that the combination of L-carnitine and coenzyme Q10 can enhance health, antioxidant capacity, milk production, and dairy product quality [17]. Studies on meat sheep have demonstrated that L-carnitine supplementation can regulate lipid metabolism, improve meat sensory quality and fat distribution [18], as well as positively influence lamb weight gain, meat performance, gene expression, and metabolic indicators [19]. Variations in the effects of L-carnitine based on species and context emphasize the need for further research to elucidate its mechanisms and optimize supplementation strategies.

Delgado et al. provided a comprehensive review highlighting the crucial role of carnitine in regulating energy metabolism and cardiovascular protection in animals, emphasizing its significant scientific value in meat improvement and health attribute optimization [20]. Studies have shown a significant correlation between carnitine content in muscles and rates of fatty acid oxidation [21,22,23] and exercise capacity [24]. In rodent models, increasing muscle carnitine levels significantly enhanced fatty acid oxidation utilization and improved exercise endurance [25]. The European Food Safety Authority [26] has officially recognized the positive effect of L-carnitine in accelerating muscle fatigue recovery after exercise, providing a solid scientific basis for its use as a health functional additive. This also opens up new research perspectives on exploring its potential mechanisms in regulating meat quality and antioxidant defense functions.

Research has demonstrated the antioxidant properties of L-carnitine as a novel nutritional supplement [27,28]. It effectively regulates animal fat metabolism [5] and enhances energy supply [1]. Previous studies have extensively explored its benefits in pigs, poultry, and aquatic animals [29], showing improvements in growth performance [30,31], meat quality [32], and disease resistance [33]. However, limited research exists on its effects in sheep. This study aims to investigate the antioxidant mechanisms of L-carnitine in the lactate dehydrogenase system and high-iron myoglobin reduction system to elucidate its role in sheep meat color formation. The findings of this research offer a theoretical foundation for incorporating L-carnitine into sheep feed and advancing the use of sustainable additives in modern sheep farming practices.

## 2. Materials and Methods

### 2.1. Animal Trials and Ration

In this study, 24 weaned wether lambs of uniform body condition and similar weight at three months of age, belonging to the F1 generation of a cross-breed between small-tailed Han sheep and Charollais sheep, were selected as experimental subjects. The experiment was conducted at the livestock farm of Shenyang Agricultural University in Shenyang City. The animals were immunized and dewormed, housed in a barn feeding system with ad libitum access to water, and the cleanliness of the barn was maintained. All experimental procedures were approved by the Animal Experiment Management Committee of Shenyang Agricultural University (approval number: IACUC-2018120101). Following the “Chinese Meat Sheep Feeding Standards” (2004) [34] and the “Chinese Feed Composition and Nutritional Value Table”, dried corn stover was used as roughage, supplemented with pelleted concentrate feed at a ratio of 7:3. The daily feed intake was calculated at 4% of body weight, with adjustments made according to the growth dynamics (the formula and nutrition level are shown in Table 1). Total mixed ration (TMR) feeding was employed, and L-carnitine with a purity of ≥99% was used. Employing a single-factor repeated complete random design, the 24 lambs were divided into three groups, each consisting of 8 F1 generation lambs with 1 lamb per replicate: a control group (Group C, basic diet only), a low-concentration L-carnitine group (Group L, basic diet + 0.01% L-carnitine), and a high-concentration L-carnitine group (Group H, basic diet + 0.05% L-carnitine). The lambs were individually housed in metal pens with separate feed troughs and waterers to allow for independent feeding and to prevent any interference. A 15-day pre-feeding period was succeeded by a formal experimental period lasting 45 days.

### 2.2. Collection and Documentation of Samples

During the trial, the daily feed intake of lambs was monitored by weighing both the feed provided and the feed refused. The methodology involved weighing and recording the dispensed feed at 08:00 daily, collecting and weighing the leftover feed after 24 h, and calculating the difference as the actual daily feed intake. The weight was measured on days 0, 15, 30, and 45 of the experiment in the morning on an empty stomach to calculate the average daily weight gain. Simultaneously, blood samples were collected from the jugular vein using vacuum anticoagulant tubes, centrifuged at 3000 rpm for 10 min to separate the plasma, which were then stored at −80 °C for analysis. Feed was withheld 24 h before the end of the trial, with a 2 h water deprivation period. After slaughter, blood was collected, animals were weighed, and relevant indicators were measured. Muscle samples were taken from the Longissimus dorsi muscle, triceps brachii, and biceps femoris [35], labeled, and stored in liquid nitrogen or at −80 °C for later analysis [36].

### 2.3. Sample Laboratory Analysis

#### 2.3.1. Quantification of L-Carnitine in Blood

The plasma L-carnitine content was quantified using an ELISA kit from Nanjing Camelot Biotechnology Co., Ltd. (Nanjing, China). Standard lyophilized samples were reconstituted, centrifuged, and mixed according to the kit instructions. Standard solutions were serially diluted in labeled centrifuge tubes, with the seventh tube serving as a negative control. Aseptic techniques were used to dispense 100 μL of sheep L-carnitine standard solution and serum samples (in triplicate) into designated wells on a plate, followed by incubation at 37 °C for 90 min. The wash buffer was diluted at a 1:25 ratio, and the biotinylated antibody was diluted at a 1:100 ratio with a specified antibody diluent. After the first incubation, the wells were washed twice. Then, 100 μL of the biotinylated antibody working solution was added and incubated at 37 °C for 60 min.. The plate was then washed five times, after which 100 μL of the enzyme conjugate working solution was added and incubated at 37 °C for 30 min. Following another incubation and washing, a TMB substrate working solution was freshly prepared by mixing solutions A and B at a 9:1 ratio (*v*/*v*). Then, 100 μL of the TMB solution was added to each well and incubated at 37 °C until a obvious color gradient appeared in the standard curve wells (approximately 20 min). Finally, 100 μL stop solution was added, and absorbance was measured at 450 nm within 10 min.

#### 2.3.2. Carcass Performance Assessment

The carcass performance assessment methods included (1) removal of the skin, hair, head, forelimbs below the carpal joint, hind limbs below the tarsal joint, and internal organs after slaughter, retaining only the kidneys and kidney fat, followed by weighing of the remaining parts after 30 min of rest; (2) measurement of backfat thickness at the midpoint above the 12th and 13th ribeye muscles using a caliper; (3) determination of the ribeye area via cross-sectioning at the posterior edge of the 12th rib on the left or right side, covering the cross-section with sulfuric paper, outlining the muscle, and calculating the area. (4) The GR value was ascertained by measuring tissue thickness between the 12th and 13th ribs at a distance of 11 cm from the spinal column using a caliper after slaughter.

#### 2.3.3. Determination of Meat Quality in the Longissimus dorsi Muscle

A method was employed to assess the quality of the Longissimus dorsi muscle through three parameters: (1) Shear force measurement involved taking a 2 cm thick sample perpendicular to the muscle fibers, heating it to 70 °C, cooling it, and measuring shear force using a Warner–Bratzler device (G-R Manufacturing Co., Manhattan, KS, USA) [37]. (2) Muscle samples were stored at 4 °C for 24 h post mortem before determining pH values. Measurement was performed using a calibrated portable pH meter (Model: PHS-25, Shanghai INESA Scientific Instrument Co., Ltd., China, Shanghai, China). A cross-shaped incision was made on the Longissimus dorsi muscle block surface to accommodate the glass electrode insertion. The electrode was inserted carefully, ensuring no contact with fat or connective tissue, and held steady until the reading stabilized (approximately 60 s). Three stable readings were taken from different locations within the incision for each sample, and the average value was recorded [38]. (3) Water loss evaluation was performed by cutting a 2 × 2 × 2 cm^3^ muscle sample, recording its initial weight, suspending it vertically in a plastic bag with wire hooks, refrigerating it at 4 °C for 24 h, and then reweighing it [39].

#### 2.3.4. Evaluation of Myoglobin Levels in Various Muscle Regions Through Coloration of the Longissimus dorsi Muscle

The methods for determining muscle color and myoglobin ratio of the Longissimus dorsi muscle were conducted as follows: (1) Muscle color was assessed using a Konica Minolta CR400 handheld colorimeter (Konika Minolta, Tokyo, Japan), calibrated with a white standard. Three readings of brightness (L*), yellowness (b*), and redness (a*) were taken vertically at different locations on each muscle sample. Chroma angle (H*) and chroma saturation (C*) were calculated based on the collected data using the following formulae: C* = (a*^2^ + b*^2^)^0.5^ and H* = arctan (b*/a*) [40]. (2) Myoglobin ratio was determined via spectrophotometry. Samples with a weight of 0.3 g were placed in 1.5 mL tubes (4 replicates), with 0.2 mm steel beads added. A volume of 1 mL of 0.04 mol/L sodium phosphate buffer was used for tissue disruption. After homogenization and centrifugation at 5000 rpm for 15 min, supernatant absorbance was measured at 525 nm, 545 nm, 565 nm, and 572 nm. Metmyoglobin (MMb) (%), oxymyoglobin (OMb) (%), and deoxymyoglobin (DMb) (%) were calculated using specific formulae involving absorbance ratios [41].

#### 2.3.5. Measurement of the Longissimus dorsi Muscle Oxygenation Index

The Longissimus dorsi muscle tissue was homogenized and subjected to high-speed centrifugation (6000 rpm, 15 min) for processing, followed by the determination of oxidative indicators in the supernatant. The MetMbase ELISA kit from Nanjing Camilo Biotechnology Co., Ltd. (Nanjing, China) was utilized following the manufacturer’s instructions, in conjunction with ELISA assay kits for MRA, SOD, LDH, and NADHB5R from Jiangsu Baolai Biotechnology Co., Ltd. (Nanjing, China), along with the BCA protein quantification kit, strictly following the protocols provided for each kit.

#### 2.3.6. Measurement of Gene Expression Associated with Flesh Color and Oxidation in the Longissimus Dorsi Muscle

Muscle tissue powder was mixed with Trizol lysis reagent (Beijing Solarbio Science & Technology Co., Ltd., Beijing, China), followed by ice incubation and centrifugation. The supernatant was collected, mixed with pre-cooled chloroform, incubated on ice, and centrifuged. After repeated chloroform treatments, 350 μL of the supernatant was transferred to an RNase-free centrifuge tube. RNA extraction involved adding isopropanol, ice incubation, centrifugation, discarding the supernatant, washing three times with 75% ethanol, centrifugation, air-drying on filter paper, and storage in DEPC water. Agarose gel electrophoresis was performed by dissolving 1 g of agarose in 100 mL of 1× TAE buffer with GoldView dye (Beijing Solarbio Science & Technology Co., Ltd., Beijing, China). Electrophoresis was run at 120 V for 20–30 min followed by gel imaging. RNA purity was assessed by the 260/280 ratio falling within 1.8–2.0. For reverse transcription, Novozymes HiScript^®^ II kit (Nanjing Vazyme Biotech Co., Ltd., Nanjing, China) was used at 42 °C for 2 min, 50 °C for 15 min, and 85 °C for 5 s to generate cDNA. Quantitative PCR (qPCR) was conducted using Novozymes ChamQ™ SYBR^®^ kit (Nanjing Vazyme Biotech Co., Ltd., Nanjing, China) with an initial denaturation at 95 °C for 3 min, followed by 40 cycles of amplification (95 °C for 15 s, 60 °C for 1 min).The primer sequences for all target genes and the housekeeping gene glyceraldehyde-3-phosphate dehydrogenase (*GAPDH*) [42] are listed in Table 2. Data analysis was performed using the 2^−ΔΔCT^ method.

#### 2.3.7. Statistical Analysis

The results are presented as mean ± SEM from a minimum of three independent experiments. Statistical analysis was performed using IBM SPSS Statistics version 12. The impact of L-carnitine supplementation was evaluated through one-way ANOVA, with the treatment group as the main factor. Upon achieving a significant overall effect (*p <* 0.05) from ANOVA, post hoc multiple comparisons were conducted using Duncan’s multiple range test to assess variances between specific groups and control for type I error in multiple comparisons. For experiments involving two groups, the two-tailed Student’s t-test was employed, and correlations were examined using Pearson’s coefficient. The significance level was set at *p <* 0.05 or *p <* 0.01.

## 3. Results

### 3.1. Effect of Dietary L-Carnitine Supplementation on the L-Carnitine Concentration in the Blood of Sheep

The analysis of the data shown in Table 3 revealed that on the 15th day, there was no statistically significant difference in the levels of L-carnitine in the plasma of sheep (*p =* 0.2136). In contrast, on the 30th day, both groups receiving low and high doses of L-carnitine showed a notable increase in L-carnitine levels compared to the control group (*p =* 5.2 × 10^−6^). By the 45th day, the group administered with a low dose exhibited significantly elevated L-carnitine levels relative to the control group (*p =* 0.0129), whereas the high-dose group not only exceeded the low-dose group (*p =* 0.013) but also demonstrated highly significant differences to the control group (*p =* 3.15 × 10^−5^).

### 3.2. Effect of L-Carnitine in the Diet on the Growth of Sheep

Through a feeding trial, the impact of dietary supplementation of L-carnitine on the growth performance of 3-month-old sheep was assessed. The results showed in Table 4 that the supplementation of L-carnitine did not produce a notable effect on the initial weight (*p =* 0.9149) or final weight (*p =* 0.9568). Nevertheless, there was a small increase in the average daily feed intake (*p =* 0.9512), with no statistically significant differences found in the average daily gain (*p =* 0.8056) and feed conversion ratio (*p =* 0.4181).

### 3.3. Effect of L-Carnitine Supplementation in the Diet of Sheep on Carcass Traits and Meat Quality

According to the data presented in Table 5, the addition of L-carnitine to the diet had no statistically significant impact on slaughter performance indicators in sheep (including carcass weight, backfat thickness, loin muscle area, GR value, and shear force) (*p >* 0.05). However, the group supplemented with L-carnitine showed a decreasing trend in backfat thickness, loin muscle area, GR value, and shear force. The pH value in the L-carnitine treatment group was closer to the standard for high-quality meat and significantly different from the control group (*p =* 0.0363). The water loss rates in the low- and high-supplementation groups were significantly lower than in the control group (*p =* 0.0228). The carcass weight, backfat thickness, and loin muscle area in the low- and high-L-carnitine groups were lower than in the control group, possibly due to L-carnitine acting as a promoter of fat metabolism, exerting a significant inhibitory effect on lamb fat deposition.

### 3.4. The Impact of Dietary L-Carnitine Supplementation on the Meat Pigmentation of Various Muscle Segments in Sheep

According to the results presented in Table 6, the a* values for redness in both the low- and high-L-carnitine treatment groups were significantly higher than those in the control group (*p =* 1.32 × 10^−5^), with saturation also being significantly higher (*p =* 0.0128). However, there were no significant differences in the b* yellowness values (*p =* 0.2675) and L* lightness values (*p =* 0. 7514). While the chroma angle in the low-dose L-carnitine group showed a decreasing trend, it did not reach statistical significance (*p =* 0.4340), whereas the chroma angle in the high-dose group was significantly lower than that in the control group (*p =* 0.004).

**Figure 1:**
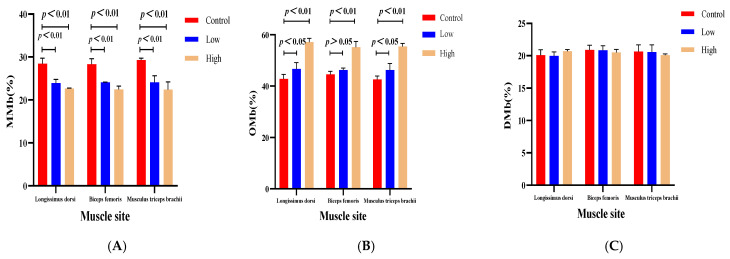
The impact of L-carnitine intervention on myoglobin metabolism in three muscle types of sheep (Longissimus dorsi, biceps femoris, musculus triceps brachii). Note: Panel (**A**) compares the metmyoglobin (MMb) proportions; Panel (**B**) analyzes the oxymyoglobin (OMb) proportions; and Panel (**C**) presents deoxymyoglobin (DMb) proportions. Each subfigure includes the control group, low-dose group, and high-dose group, providing a comprehensive display of the varied effects of L-carnitine supplementation.

The study measured the myoglobin content in the Longissimus dorsi, biceps femoris, and triceps brachii muscles of sheep. The results (Table 7) showed that dietary supplementation with L-carnitine significantly increased the myoglobin content in sheep muscles, with the highest content observed in the high-dose L-carnitine group (*p <* 0.01). Specifically, in the Longissimus dorsi muscle of sheep in the low- and high-dose L-carnitine groups, the OMb content increased by 4.59% and 15.01% (*p =* 0.0002), while the MMb content decreased by 4.53% and 5.73% (*p =* 0.0005) (Table 8 and Figure 1). In the biceps femoris, the OMb content increased by 1.65% and 10.53% (*p =* 0.0003), while the MMb content decreased by 5.18% and 5.85% (*p =* 0.0005). In the triceps brachii, the OMb content increased by 3.72% and 12.86% (*p =* 0.0003), while the MMb content decreased by 5.41% and 5.74% (*p =* 0.0025). No significant difference in DMb content was observed across all three sites (*p =* 0.3675, *p =* 0.7519, *p =* 0.7263). The findings suggest that L-carnitine supplementation could effectively reduce MMb content in sheep muscles while increasing OMb content.

### 3.5. Effects of Dietary Inclusion of L-Carnitine on the Oxidation Reduction Status of the Longissimus Dorsi Muscle in Hybrid Sheep

According to Table 9, the metmyoglobin reductase activity (MRA) in the low- and high-concentration L-carnitine groups was significantly increased compared to the control group (*p =* 3.27 × 10^−5^). Additionally, the levels of lactate dehydrogenase (LDH), superoxide dismutase (SOD), and NADH-cytochrome b5 reductase in the longest muscle of sheep back in the low- and high-concentration L-carnitine groups were significantly higher than those in the control group (*p <* 0.01), indicating that the addition of L-carnitine can enhance the antioxidant capacity in muscles.

### 3.6. The Relationship Between Concentrations of L-Carnitine (45 d) and Biochemical Indicators

According to the data analysis in Table 10, the concentrations of L-carnitine (45 d) showed a significant positive correlation with the redness value a* (r = 0.912, *p <* 0.01), as well as with MRA activity (r = 0.939, *p <* 0.01) and myoglobin content (r = 0.950, *p <* 0.01). L-carnitine concentration also exhibited a significant negative correlation with MMb proportion (r = −0.874, *p <* 0.01) and a significant positive correlation with OMb proportion (r = 0.974, *p <* 0.01). High-iron myoglobin reductase activity was significantly positively correlated with the a* value (r = 0.943, *p <* 0.01) and significantly negatively correlated with MMb proportion (r = −0.961, *p <* 0.01) and positively correlated with OMb proportion (r = 0.891, *p <* 0.01). The a* value was highly negatively correlated with MMb proportion (r = −0.917, *p <* 0.01) and highly positively correlated with OMb proportion (r = 0.863, *p <* 0.01). MMb proportion was highly negatively correlated with OMb proportion (r = −0.796, *p <* 0.01). High-iron myoglobin reductase activity was significantly positively correlated with lactate dehydrogenase (r = 0.582, *p <* 0.05) and cytochrome b5 reductase (r = 0.547, *p <* 0.05). Superoxide dismutase activity was significantly positively correlated with lactate dehydrogenase (r = 0.783, *p <* 0.01) and cytochrome b5 reductase (r = 0.938, *p <* 0.01) and significantly negatively correlated with MMb proportion (r = −0.628, *p <* 0.05). LDH and NADHBR5 were both highly negatively correlated with MMb proportion (r = −0.717, *p <* 0.01; r = −0.682, *p <* 0.01), indicating that the addition of L-carnitine enhanced MRA activity, thereby directly affecting muscle color toward higher quality. Additionally, there was an association between high-iron myoglobin reductase, lactate dehydrogenase, and cytochrome b5 reductase, suggesting an interrelation within the lactate dehydrogenase redox system.

### 3.7. Assessment of mRNA Expression Levels of Genes Involved in Oxidation Reduction and Meat Color Formation

**Figure 2:**
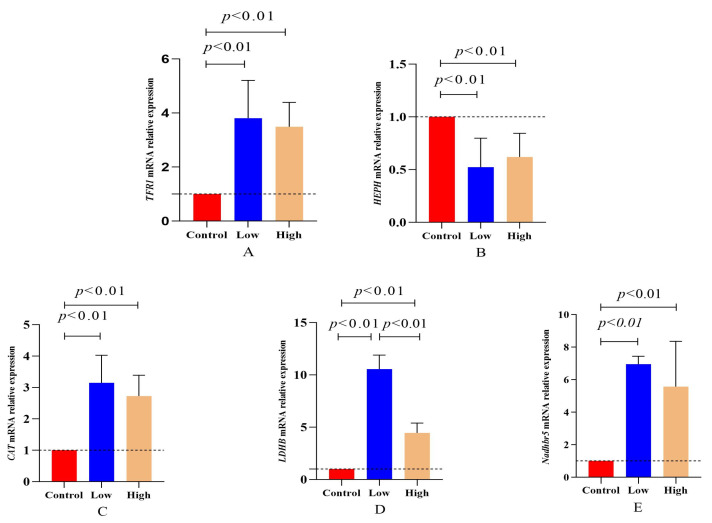
Effects of L-carnitine on mRNA expression of genes related to oxidation reduction reactions in the Longissimus dorsi muscle of cross-bred sheep. Note: (**A**): *TFR1*, (**B**): *HEPH*, (**C**): *CAT*, (**D**): *LDHB*, and (**E**): *NADHBR5*. The groups were labeled as Control for the control group, Low for the low-dose of L-carnitine group, and High for the high-dose of L-carnitine group. Gene expression levels were normalized to the control group (set as 1). The dashed line indicates the baseline expression level of the control.

Iron ions and ferrous ions were involved in the expression of transmembrane transport proteins *TFR1* and *HEPH*, regulating the ion balance. The results presented in Figure 2A,B show that in the low- and high-dose L-carnitine groups, *TFR1* mRNA expression was significantly higher than that in the control group (*p =* 1.25 × 10^−5^), while *HEPH* mRNA expression was significantly lower than that in the control group (*p =* 1.61 × 10^−4^), indicating higher Fe^2+^ levels than Fe^3+^ in lamb muscle tissue. Figure 2C–E demonstrate that in the L-carnitine group, the mRNA expressions of *CAT*, *LDHB*, and *NDHB5R* were all significantly higher than those in the control group (*p <* 0.01), confirming that L-carnitine supplementation enhanced the body’s antioxidant capacity and the function of the lactate dehydrogenase redox system.

## 4. Discussion

L-carnitine, a key molecule in fat oxidation and energy metabolism, plays a crucial role in lipid metabolism in animals [5,43]. Studies showed that supplementation of exogenous L-carnitine could effectively improve muscle bioenergetic status, reduce fat accumulation, and enhance exercise endurance [44,45]. While further research is needed in the field of ruminant animal nutrition, existing evidence suggests its positive effects in regulating fat metabolism and energy utilization, potentially optimizing animal meat quality and economic benefits [46,47]. The preliminary results of this experiment indicated significant temporal and dose-dependent changes in plasma L-carnitine levels in hybrid sheep, suggesting that its supplementation effects were subject to specific conditions [48,49]. Although L-carnitine did not significantly accelerate the growth rate of hybrid sheep, it may have indirectly improved the absorption and utilization of nutrients by adjusting energy metabolism pathways [50], particularly in regulating fat deposition. The study found that the addition of L-carnitine had no statistically significant impact on the slaughter performance indicators of hybrid sheep. However, the muscle pH values in the L-carnitine-treated group were closer to the standards of high-quality meat. The water loss rates in the low- and high-dosage groups were significantly lower than those in the control group, indicating that L-carnitine supplementation, while not significantly improving growth rates, had a certain effect on enhancing meat quality and reducing fat deposition. A significant increase in pH values was observed in the high-L-carnitine group (0.05% L-carnitine), which may play a key role in mediating improvements in other meat quality traits, particularly the reduction in drip loss. The water-holding capacity of meat is greatly influenced by its final pH value [51,52]. Higher pH values lead muscle proteins away from their isoelectric point, increasing net charge and enhancing water-holding capacity [53]. Therefore, the significant reduction in drip loss in the high-L-carnitine group (0.05% L-carnitine) compared to the control group (36.12% vs. 39.22%, *p <* 0.05) can at least partially be attributed to its significantly higher pH value (6.26 vs. 5.94, *p <* 0.05). While L-carnitine may have direct impact on post-slaughter cell membrane integrity and energy metabolism, this pH-mediated effect provides a fundamental biochemical explanation for the observed improvement in water-holding capacity. Future research focusing on measuring glycogenolytic potential and protein phosphorylation can further elucidate the precise mechanisms by which L-carnitine supplements influence post-slaughter pH decline. In terms of muscle color, the inclusion of L-carnitine in the diet significantly increased the redness (a* value) and color saturation of the muscle, making the meat color closer to that of fresh high-quality meat. Furthermore, L-carnitine increased the total myoglobin content in the muscles of hybrid sheep and significantly reduced the relative proportion of high-iron myoglobin in the Longissimus dorsi, biceps femoris, and triceps brachii, further confirming the potential mechanism by which L-carnitine indirectly improved meat color by regulating the oxidation reduction state of iron ions.

In terms of growth performance, no significant impact of L-carnitine on average daily weight gain was observed in this study, possibly due to multiple factors, such as the breed and age of the experimental sheep [54], as well as the proportion of L-carnitine added [55]. The differences in results under different experimental conditions suggested that when using L-carnitine as a feed additive, the individual differences in animals, dosage design, and the interactive effects of the feeding environment should be fully considered. From a mechanistic perspective, the content and storage form of myoglobin in muscles directly affected meat color [56]. The role of L-carnitine in increasing myoglobin content and improving muscle color was closely related to its regulation of high-iron myoglobin reductase, further elucidating the multiple roles of L-carnitine in regulating muscle biochemical composition [57]. Previous studies had indicated that the formation of muscle color was influenced by the oxidation states of iron ions, with the ratio of Fe^2+^ to Fe^3+^ determining the oxidation state of myoglobin and the final color presented [58]. The decrease in the proportion of high-iron myoglobin due to L-carnitine in the experiment may have suggested its optimization of muscle color by regulating the iron ion environment and promoting the reduction in high-iron myoglobin.

Previous studies had confirmed the significant role of L-carnitine in promoting the antioxidative process in animal organisms [59]. L-carnitine enhanced energy conversion efficiency by reducing tissue lipid content and promoting fat metabolism [60]. Research by Wakil et al. had demonstrated that the concentration of L-carnitine directly influenced fatty acid β-oxidation capacity [61], with higher concentrations accelerating fatty acid translocation across the mitochondrial inner membrane [62], thereby enhancing mitochondrial metabolic capacity [63]. Oxidative stress reactions could overwhelm the body’s metabolic system with an excess of highly reactive molecules (reactive oxygen, reactive nitrogen), leading to oxidative system imbalance and tissue damage [64]. L-carnitine effectively scavenged excess reactive oxygen species or inhibited their production, thereby resisting oxidative stress damage and preventing lipid peroxidation reactions [65]. The a* value, a key parameter for assessing the redness saturation of meat products, was closely related to the oxygenation state of myoglobin [59].

The results of this study demonstrated a significant positive correlation between the concentration of L-carnitine added and the a* value. Additionally, the a* value showed a significant negative correlation with the relative proportions of metmyoglobin and a significant positive correlation with the proportion of oxymyoglobin [66], indicating that dietary L-carnitine, particularly at the 0.05% level, optimized meat color by regulating the oxidation reduction state of myoglobin. In the L-carnitine-treated group, the expression of SOD was significantly higher compared to the control group. SOD, as a crucial antioxidant enzyme, scavenges superoxide anions (O^2−^) by catalyzing their dismutation into H_2_O_2_ [67] and oxygen, effectively preventing the chain reactions initiated by O^2−^ [68]. Both low- and high-L-carnitine treatment groups exhibited significantly higher levels of CAT expression compared to the control group. *CAT*, as a peroxidase with H_2_O_2_ as a substrate, converted H_2_O_2_ to water, efficiently eliminating excess H_2_O_2_ produced by SOD catalysis of O^2−^ [69], thereby protecting tissue cells [70]. Furthermore, ferrochelatase catalyzed the chelation of ferrous ions (Fe^2+^) with protoporphyrin, forming iron protoporphyrin favorable for cytochrome electron transfer in cells [71]. The study findings suggested that L-carnitine supplementation could enhance SOD enzyme activity and *CAT* expression levels in the early-growth-stage muscles of weaned lambs, boosting the body’s antioxidant capacity, facilitating the catalysis of excess reactive oxygen species into H_2_O_2_, and ultimately converting them into water. This implied the presence of an antioxidant redox reaction electron transfer chain in lamb muscles. The results demonstrated that while both concentrations increased MRA activity compared to the control, the high concentration (0.05%) resulted in a significantly greater increase than the low concentration (0.01%), confirming the effectiveness of L-carnitine in enhancing the antioxidant capacity of sheep muscles. It also contributed to redox homeostasis by enhancing the clearance of superoxide anions (O^2−^) and H_2_O_2_ through the elevated activity of SOD and CAT [72]. The high-iron myoglobin reduction system was a crucial biochemical mechanism for maintaining the color of meat, involving the reduction of oxidized high-iron myoglobin (Fe^3+^) to its oxygen-binding form, ferrous myoglobin (Fe^2+^) [73]. TFR1 and HEPH were the iron transport receptor proteins that served as upstream regulatory genes of hepcidin, reflecting the body’s iron demand regulation mechanism [74,75]. Studies showed that under severe oxidative stress, excessive accumulation of Fe^3+^ led to a significant upregulation of HEPH [76]. Following L-carnitine treatment, TFR1 was significantly upregulated, while HEPH was markedly downregulated, indicating that L-carnitine inhibited the excessive oxidation of divalent iron ions by O^2−^ to trivalent iron ions in lamb muscles, reducing the Fe^3+^ content in muscle tissues and thereby suppressing the relative proportion increase in high-iron myoglobin [77]. These findings suggested that supplementation with L-carnitine could enhance the activity of high-iron myoglobin reductase in the Longissimus dorsi muscle of early-growing sheep, thereby improving the color of lamb meat.

During cellular redox processes, most oxidoreductases utilized nicotinamide adenine dinucleotide (NADH/NAD^+^) as a coenzyme to facilitate electron transfer between substrates and enzymes [78]. A few oxidoreductases utilized coenzymes such as nicotinamide adenine dinucleotide phosphate (NADPH/NADP^+^), flavin adenine dinucleotide (FAD), and flavin mononucleotide (FMN) [79]. High-iron myoglobin reductase, dependent on NADH, consisted of a reduction system in the mitochondrial membrane involving NADH, cytochrome b5, and cytochrome b5 reductase. NADH oxidases, including H_2_O-forming, H_2_O_2_-forming, and mixed-type enzymes, played crucial regulatory roles in energy metabolism and antioxidant stress [80]. They directly reduced O_2_ to H_2_O or H_2_O_2_ through electron transfer and regenerated NAD^+^ from NADH [81]. The increased activity of high-iron myoglobin reductase may have reflected elevated NADH levels in the mitochondrial membrane. L-carnitine participated in energy metabolism within the mitochondrial membrane, influencing metabolic efficiency and the rate of redox reactions in the electron transport chain [46]. Various dehydrogenases affected meat color stability in energy metabolism [82,83]. Studies indicated that LDH activity partly reflected the activity of catalyzing product NADH, a key factor in the high-iron myoglobin reduction process.

The results of this experiment demonstrated that supplementing lamb feed with L-carnitine significantly increased the activity of lactate dehydrogenase and the expression level of *LDHB* mRNA in lamb muscles. This indicated that L-carnitine supplementation enhanced the oxidative conversion of lactate to pyruvate and electron transfer within cells, effectively eliminating excess reactive oxygen species. *NADHB5R* played a crucial role in the electron transfer process of the cellular respiratory chain by catalyzing the transfer of hydrogen atoms from NADH to the FAD coenzyme, facilitating the reduction of the iron atom in ferroheme proteins. Previous studies had shown that in the mitochondrial membrane, NADH served as the initial electron donor, transferring electrons through the electron transport chain to cytochrome b5 reductase [84], which then passed them to cytochrome b5 [85]. The reduced cytochrome b5 subsequently transferred electrons to the heme prosthetic group of high-iron myoglobin, leading to the reduction of Fe^3+^ to Fe^2+^ [86]. The results of this experiment indicated that L-carnitine significantly enhanced the activity of NADH-cytochrome b5 reductase and the expression level of *NDHB5R* in lamb muscles, promoting the transfer of NADH hydrogen atoms and the reduction of iron ions in cytochrome b5 ferroheme proteins, thus delaying the rate of meat discoloration [18]. Treatment with L-carnitine significantly upregulated the mRNA expression of *CAT*, *LDHB*, and *NDHB5R* compared to the control group, confirming its promotion of antioxidant capacity and lactate dehydrogenase redox system function in the body [28]. Catalase rapidly breaks down hydrogen peroxide in cells, protecting them from oxidative damage [87]. The upregulation of *LDHB* and *NDHB5R*, which are the key players in lactate and energy metabolism, may have enhanced metabolic efficiency and thereby alleviated oxidative stress. The antioxidant capacity, tissue cell mitochondrial activity, and electron transfer rate all impacted muscle coloration [88]. L-carnitine significantly regulated early muscle coloration in stable sheep growth. Supplementation of L-carnitine in diet significantly modulated early muscle metabolism in lambs [89], enhancing the muscle tissue’s redox system by activating lactate dehydrogenase and NADH-cytochrome b5 reductase. This physiological mechanism not only promoted electron transfer [46] and high-valence iron reduction but also significantly increased high-iron myoglobin reductase activity, reducing the proportion of high-iron myoglobin and favoring oxy-myoglobin synthesis, delaying undesirable changes in muscle coloration. While these mechanistic findings are clear, it should be noted that the use of L-carnitine is not without its limitations, as its efficacy can be influenced by factors such as individual metabolic variations, dietary intake, and the timing of supplementation, which may affect its overall impact on muscle quality.

## 5. Conclusions

L-carnitine supplementation at the 0.05% level significantly improved the quality of the Longissimus dorsi muscle in hybrid sheep. The research demonstrated that this additive effectively reduced muscle drip loss, increased the meat pH value, and brought the meat quality closer to the optimal standard. L-carnitine not only significantly enhanced the redness value of the Longissimus dorsi muscle but also showed a close association with the redox status of myoglobin. This improvement was primarily driven by a significant increase in MRA activity. By elevating the levels of lactate dehydrogenase and cytochrome b5 reductase, L-carnitine enhanced the antioxidant and redox capacity within the muscle, optimized muscle color, and thereby most effectively improved the overall quality of sheep meat.

## Figures and Tables

**Table 1:** Composition and nutrient levels of the basal diet (%, DM basis).

Ingredients	Composition (%)	Nutritive Index	Nutrient Levels
Corn	40.0	Dry matter, %	87.70
Soybean meal	17.0	Net energy, MJ/kg	5.84
DDGS	8.0	Crude protein, %	18.50
Corn germ meal	6.2	Crude fat, %	3.50
Rice bran meal	6.0	Crude fiber, %	6.20
Puffed corn	5.0	Crude ash, %	5.90
Soybean skin	4.0	Calcium, %	1.10
Beet pulp pellet	4.0	Total phosphorus, %	0.50
Puffed soybean powder	3.0	Lysine, %	1.25
Fermented soybean meal	3.0	Methionine, %	0.48
Mountain flour	2.2	Met + Cys, %	0.83
Salt	0.6	Threonine, %	0.66
Compound premix ^1^	1.0	Tryptophan, %	0.20
Total	100.0		

Note: ^1^ Premix provides the following per kilogram of feed: copper 450–620 mg, manganese 800–1300 mg, zinc 1000–1800 mg, iron 800–1500 mg, iodine 5.0–25.0 mg, selenium 2.0–12.0 mg, cobalt 5.0–25.0 mg, vitamin A acetate 10.0–50.0 × 10^4^ IU, vitamin D3 2.00–10.0 × 10^4^ IU, and folic acid ≥ 400 mg.

**Table 2:** Primer design.

Official Symbol	GenBank ID	Primer Sequences (5′–3′)	Amplified Fragment Size (bp)	Annealing Temperature (°C)
*HEPH*	101113655	5′-TTTATTCTCAACCAAGGCTAT-3′ 5′-GGAAATAAGTAGGAGTGAGGTT-3′	134	48
*NADHB5R*	101123639	5′-ACAGACAACGCAGAGGACGAG-3′ 5′-CTGGCTGAGGACAAAGAAGGT-3′	135	58
*CAT*	100307035	5′-TGCTCTACTGTTTCCGTCCTT-3′ 5′-GAACGGATATGGATCGCATAC-3′	186	60
*LDHB*	101118315	5′-GTCCGTTGTGGATCTGACCTG-3′ 5′-GTGTAGCCTAGAATGCCCTTG-3′	111	55
*TFR1*	100885768	5′-CAAAGTTTCTGCCAGTCCGC-3′ 5′-GATCCAGTTGCTGTCCCGAT-3′	106	58
*GAPDH*	443005	5′-TTTATTCTCAACCAAGGCTAT-3′ 5′-GGAAATAAGTAGGAGTGAGGTT-3′	78	55

**Table 3:** Concentration of L-carnitine in blood of sheep at different times.

Days	L-Carnitine (ng/mL)			SEM	*p*-Value		
	0 (C)	0.01% (L)	0.05% (H)		C:L	C:H	L:H
15	46.89	47.69	45.76	4.30	0.85	0.80	0.68
30	48.88	53.92	52.98	1.47	<0.05	<0.05	0.95
45	52.53	56.79	60.41	1.65	<0.05	<0.01	<0.05

Note: C = Control level of L-carnitine; L = Low level of L-carnitine; H = High level of L-carnitine; SEM = Standard error of means (each treatment mean represents three replicates).

**Table 4:** Effect of L-carnitine supplementation in the diet on the growth of sheep.

Items	L-Carnitine (Gram/Sheep/Day)			SEM	*p*-Value		
	0 (C)	0.01%(L)	0.05% (H)		C:L	C:H	L:H
Initial Wt. (kg)	15.53	14.93	15.39	1.48	0.69	0.92	0.76
Final Wt. (kg)	17.55	16.95	16.94	2.37	0.80	0.79	0.99
ADFI (kg)	0.4482	0.4684	0.4606	0.0109	0.12	0.30	0.50
ADG (kg)	0.0669	0.0667	0.0671	0.0152	0.29	0.68	0.50
F/G	6.71	6.82	6.81	0.19	0.67	0.87	0.11

**Table 5:** Effect of L-carnitine supplementation in the diet on the carcass traits and meat quality of sheep.

Items	L-Carnitine (Gram/Sheep/Day)			SEM	*p*-Value		
	0 (C)	0.01% (L)	0.05% (H)		C:L	C:H	L:H
Carcass weight (kg)	8.43	7.81	8.43	0.67	0.38	1.00	0.38
Backfat thickness (cm)	0.27	0.17	0.23	0.08	0.27	0.70	0.45
Loin muscle area (cm^2^)	4.13	2.63	3.49	0.92	0.15	0.51	0.39
GR value (cm)	0.40	0.28	0.32	0.11	0.28	0.78	0.41
Shear force (N)	5.38	4.68	4.84	0.86	0.45	0.55	0.86
pH value	5.94	6.03	6.26	0.11	0.47	<0.05	0.08
Drip loss (%)	39.22	35.71	36.12	0.98	<0.05	<0.05	0.69

**Table 6:** Influence of dietary inclusion of L-carnitine on the color of the Longissimus dorsi muscle in sheep.

Items	L-Carnitine (Gram/Sheep/Day)			SEM	*p*-Value		
	0 (C)	0.01% (L)	0.05% (H)		C:L	C:H	L:H
a* value	16.88	18.06	19.07	0.40	<0.01	<0.01	<0.05
b* value	10.74	10.98	10.04	0.58	0.69	0.24	0.12
L* value	37.51	38.67	38.37	1.89	0.47	0.59	0.85
Meat color saturation	20.04	21.14	21.58	0.49	<0.05	<0.05	0.38
Chroma angle	0.56	0.54	0.48	0.02	0.43	<0.01	<0.05

**Table 7:** The effects of carnitine supplementation in the diet on myoglobin concentrations in different muscle regions of sheep.

Items	Myoglobin Concentrations (mg/g)			SEM	*p*-Value		
	0 (C)	0.01% (L)	0.05% (H)		C:L	C:H	L:H
Longissimus dorsi muscle	2.84	3.29	3.52	0.07	<0.01	<0.01	<0.05
Biceps femoris	2.90	3.34	3.55	0.05	<0.01	<0.01	<0.05
Triceps brachii	2.98	3.28	3.60	0.06	<0.01	<0.01	<0.05

**Table 8:** The effect of carnitine dietary supplementation on the proportion of three distinct myoglobin oxidation forms across different sheep muscle types.

Items		Relative Concentration Ratio (%)			SEM	*p*-Value		
		0 (C)	0.01% (L)	0.05% (H)		C:L	C:H	L:H
Longissimus dorsi	MMb	28.46	23.93	22.66	0.72	<0.01	<0.01	0.07
	OMb	42.77	46.70	57.11	1.49	<0.05	<0.01	<0.01
	DMb	20.07	19.97	20.70	0.66	0.86	0.27	0.25
Biceps femoris	MMb	28.30	24.13	22.46	0.72	<0.01	<0.01	0.06
	OMb	44.58	46.23	55.11	1.26	0.24	<0.01	<0.01
	DMb	20.89	20.83	20.51	0.54	0.92	0.48	0.20
Musculus triceps brachii	MMb	29.27	24.07	22.40	1.03	<0.01	<0.01	0.20
	OMb	42.55	46.27	55.42	1.47	<0.05	<0.01	<0.01
	DMb	20.65	20.55	20.09	0.73	0.90	0.47	0.66

**Table 9:** Impact of L-carnitine supplementation on the antioxidant capacity of the Longissimus dorsi muscle in cross-bred sheep.

Items	L-Carnitine (Gram/Sheep/Day)			SEM	*p*-Value		
	0 (C)	0.01% (L)	0.05% (H)		C: L	C:H	L:H
SOD (U/L)	223.15	343.53	275.73	14.60	<0.01	<0.01	<0.01
LDH (U/L)	3.66	4.83	4.45	0.23	<0.01	<0.01	0.11
NADHB5R (U/L)	94.65	126.14	106.71	3.35	<0.01	<0.01	<0.01
MRA (ng/mL)	2.25	3.53	4.21	0.08	<0.01	<0.01	<0.01

**Table 10:** Pearson bivariate linear correlation analysis.

	L-Carnitine	a*	MRA	SOD	LDH	NADHBR5	Myoglobin	MMb	OMb
L-Carnitine (45d)	1								
a*	0.912 **	1							
MRA	0.939 **	0.943 **	1						
SOD	0.236	0.436	0.487	1					
LDH	0.267	0.497	0.582 *	0.783 **	1				
NADHB5R	0.377	0.482	0.547 *	0.938 **	0.834 **	1			
Myoglobin	0.950 **	0.943 **	0.978 **	0.411	0.570 *	0.472	1		
MMb	−0.874 **	−0.917 **	−0.961 **	−0.628 *	−0.717 **	−0.682 **	−0.953 **	1	
OMb	0.974 **	0.863 **	0.891 **	0.143	0.305	0.146	0.923 **	−0.796 **	1

Note: The symbols * and ** represent a significant correlation at *p* < 0.05 and an extremely significant correlation at *p* < 0.01, respectively.

## Data Availability

The article contains the information that was utilized to support the study’s conclusions.

## Abbreviations

The following abbreviations are used in this manuscript:

TMR	Total mixed ration
DM	Dry matter
DDGS	Distillers dried grains with solubles
ADFI	Average daily feed intake
ADG	Average daily gain
F/G	Feed-to-gain ratio
GR	Weight-adjusted fatness value
OMb	Oxymyoglobin
MMb	Metmyoglobin
DMb	Deoxymyoglobin
MRA	Metmyoglobin reductase activity
SOD	Superoxide dismutase
LDH	Lactate dehydrogenase
LDHB	Lactate dehydrogenase-B
NADHB5R	Nadh-cytochrome b5 reductase
H_2_O_2_	Hydrogen peroxide
CAT	Catalase
ROS	Reactive oxygen species
TRF1	Transferrin receptor 1
HEPH	Hephaestin
GAPDH	Glyceraldehyde-3-phosphate dehydrogenase
